# Rapid learning dynamics in individual honeybees during classical conditioning

**DOI:** 10.3389/fnbeh.2014.00313

**Published:** 2014-09-15

**Authors:** Evren Pamir, Paul Szyszka, Ricarda Scheiner, Martin P. Nawrot

**Affiliations:** ^1^Bernstein Center for Computational NeuroscienceBerlin, Germany; ^2^Neuroinformatics and Theoretical Neuroscience, Institute of Biology, Freie Universität BerlinGermany; ^3^Department Genetics of Learning and Memory, Leibniz Institute for NeurobiologyMagdeburg, Germany; ^4^Department of Biology, University of KonstanzKonstanz, Germany; ^5^Department of Behavioral Physiology and Sociobiology (Zoology II), University of WürzburgWürzburg, Germany

**Keywords:** *Apis mellifera*, proboscis extension response (PER), single-trial learning, classical conditioning, Rescorla–Wagner model, sucrose sensitivity, sucrose responsiveness, learning curve

## Abstract

Associative learning in insects has been studied extensively by a multitude of classical conditioning protocols. However, so far little emphasis has been put on the dynamics of learning in individuals. The honeybee is a well-established animal model for learning and memory. We here studied associative learning as expressed in individual behavior based on a large collection of data on olfactory classical conditioning (25 datasets, 3298 animals). We show that the group-averaged learning curve and memory retention score confound three attributes of individual learning: the ability or inability to learn a given task, the generally fast acquisition of a conditioned response (CR) in learners, and the high stability of the CR during consecutive training and memory retention trials. We reassessed the prevailing view that more training results in better memory performance and found that 24 h memory retention can be indistinguishable after single-trial and multiple-trial conditioning in individuals. We explain how inter-individual differences in learning can be accommodated within the Rescorla–Wagner theory of associative learning. In both data-analysis and modeling we demonstrate how the conflict between population-level and single-animal perspectives on learning and memory can be disentangled.

## Introduction

Classical conditioning relies on the assumption that changes in conditioned response (CR) probability observed during training adequately represent neuronal plasticity (Dubnau et al., 2003; Dudai, 2004). Commonly, behavioral plasticity is quantified by averaging over a population of identically treated animals. However, average performance scores can obscure the learning dynamics in individuals (Gallistel et al., 2004). Animals in a given sample can vary considerably in several attributes of individual learning, such as in the ability to learn a task, the speed of learning, and the asymptotic performance (Dukas, 2008). In the extreme case of pronounced inter-individual learning differences in the sample, the group-average account of learning will be inappropriate and may even confound the analysis at the neuronal level. To give an example: The distribution of learning capabilities in a group of trained animals may be bimodal. One proportion of animals may rapidly learn, while the other group of animals is not able to learn the task. The group-average learning asymptote will then reflect the ratio between learners and non-learners. However, it will not reflect the asymptotic learning performance of any of the animals in the sample. To give another example: Learning may progress at different speeds in different animals. One proportion of animals may learn the task in a single trial, while others require more training. Looking at the rise of the group-average learning curve will then imply a performance improvement over training trials, which for single-trial learners is not justified.

The honeybee (*Apis mellifera*) has been a valuable model to study the neuronal mechanisms of learning and memory (Menzel, 2001, 2012; Schwärzel and Müller, 2006; Giurfa, 2007; Giurfa and Sandoz, 2012). In classical conditioning of the proboscis extension response (PER) it had early on been recognized that individual honeybees can rapidly acquire an association between the olfactory stimulus and the sugar reward in as few as a single conditioning trial (Bitterman et al., 1983). However, at the same time researchers relied on group average measures in order to characterize and quantify the course of acquisition (Bitterman et al., 1983), as well as the dynamics of memory formation (Menzel, 1990). Only recently has a study pointed out that group-average behavior only provides a poor description of learning in individual honeybees during classical conditioning (Pamir et al., 2011).

Building on this study and an extended collection of behavioral data (Table 1) we here present an alternative parametric description of the data. We quantify learning by the following three behavioral parameters: (1) the percentage of non-responders in a given sample, (2) the time-point in trial time at which animals show their first CR (*t*_*firstCR*_), and (3) the stability of the CR in responding animals during consecutive trials (CR stability). Focusing on these three parameters allows us to disentangle the group-average perspective on learning from the single-animal perspective on learning. We analyzed the modulation of the three parameters for several classical conditioning protocols: absolute conditioning (one stimulus is paired with a reward) with a different number of conditioning trials, absolute conditioning with short inter-trial-intervals (massed conditioning), absolute conditioning with different inter-stimulus-intervals (trace conditioning), and differential conditioning (one stimulus is paired with reward and another stimulus is presented alone).

**Table 1:** **Overview over analyzed data from classical conditioning of the proboscis extension response**.

**Dataset**	***N***	***m***	***T(h)***	***ITI(min)***	**CS**	**Experimenter**	**References**
1	64	3	24, 48	2	Clove oil	VA, JF, DE	Pamir et al., 2011
2	58	3	24	2	Clove oil	LM, JF, DE	Pamir et al., 2011
3	87	3	24	10	Clove oil	KBG, DE	Pamir et al., 2011
4	517	3	24	10	Clove oil	NS, DE	Stollhoff et al., 2005; Pamir et al., 2011
5	98	3	25	10	Clove oil	NS, DE	Stollhoff et al., 2005; Pamir et al., 2011
6	113	3	26	10	Clove oil	NS, DE	Stollhoff et al., 2005; Pamir et al., 2011
7	92	3	28	10	Clove oil	NS, DE	Stollhoff et al., 2005; Pamir et al., 2011
8	85	3	48	10	Clove oil	NS, DE	Stollhoff et al., 2005; Pamir et al., 2011
9	94	3	72	10	Clove oil	NS, DE	Stollhoff et al., 2005; Pamir et al., 2011
10	122	4	1, 24	30	Isoamyl acetate	NKC	Pamir et al., 2011
11	37	5	1, 24	30	6-Pentadecene	NKC	Pamir et al., 2011
12	48	5	1, 24	30	7-Pentadecene	NKC	Pamir et al., 2011
13	95	6	0.25	10	1-Nonanol	PS	Szyszka et al., 2011
14	75	6	0.25	10	1-Nonanol	PS	Szyszka et al., 2011
15	281	6	0.25	10	1-Octanol or 2-heptanone	PS	Pamir et al., 2011
16	100	11	24	5	Citral	RS	Scheiner et al., 2001a
17	100	11	24	5	Tactile conditioning	RS	Scheiner et al., 2001a
18	63	12	None	0.5	Hexanol	RM	Menzel et al., 2001; Pamir et al., 2011
19	64	12	None	15	Hexanol	RM	Menzel et al., 2001; Pamir et al., 2011
20	120	6	1, 24	14	1-Hexanal, 1-octanol	NKC	Pamir et al., 2011
21	118	4	24	10	1-Hexanol or 1-nonanol	PS	Unpublished data
22	335	2–4	24	10	1-Hexanol or 1-nonanol	PS	Unpublished data
23	121	2	24	10	1-Hexanol or 1-nonanol	PS	Unpublished data
24	118	1	24	10	1-Hexanol or 1-nonanol	PS	Unpublished data
25	293	1, 3	24	10	1-Hexanol or 1-nonanol	PS	Unpublished data

Abbreviations: N, number of animals; m, number of conditioning trials in the acquisition session; T, time of the retention test in hours after the end of the conditioning session; ITI, inter-trial-interval during conditioning trials in minutes; CS, conditioned stimulus; Experimenters: VA, Victoria Antemann; JF, Johannes Felsenberg; DE, Dorothea Eisenhardt; KBG, Katrin Barbara Gehring; LM, Laura Morgenstern; NS, Nicola Stollhoff; NKC, Neloy Kumar Chakroborty; RS, Ricarda Scheiner; RM, Randolf Menzel; PS, Paul Szyszka; See Section Materials and Methods for details on individual data sets.

We also wanted to establish a better understanding of memory retention after the training phase and to identify possible confounds arising from the population-level perspective. The current model of memory phases in the honeybee distinguishes between single-trial and multiple-trial induced memories (Menzel, 1999; Müller, 2012). Behavioral evidence for this model is provided by the observation that multiple-trial conditioning generally results in high retention scores whereas single-trial conditioning produces lower retention when tested at different time points after training (Menzel, 1990, Figure 9.8). While this finding holds true at the population level, we were asking if it also holds true at the individual level, hence if indeed more training results in stronger retention in individual honeybees.

For the honeybee, several studies showed that factors such as satiation level, behavioral role or age have an effect on individual responsiveness for sucrose, which in turn affects learning performance (Scheiner et al., 1999; Friedrich et al., 2004; Behrends and Scheiner, 2012). Re-analyzing data in which the responsiveness to sucrose was estimated prior to conditioning we studied the correlation between this experimental measure and the individual learning dynamics.

Finally, we consider the consequences of our findings for the theoretical account of learning in the honeybee and explain how the well-known Rescorla–Wagner model (Rescorla and Wagner, 1972) can be applied to the behavioral data in a more informative way.

## Materials and methods

All experiments were performed with foragers of the honeybee *Apis mellifera*.

### Classical conditioning of the proboscis extension response in the honeybee

Olfactory classical conditioning of the proboscis extension response (PER) (extension of their mouthparts) in the honeybee has been described in detail (Bitterman et al., 1983; Scheiner et al., 2003a; Stollhoff et al., 2005; Felsenberg et al., 2011; Szyszka et al., 2011). Briefly, during the conditioning session, a group of *N* animals was individually exposed to *m* forward pairings of the conditioned stimulus (CS, odor) with the unconditioned stimulus (US, sucrose). Memory retention was measured by presenting the CS alone at time point *T*(*h*) after conditioning. Only animals which responded with proboscis extension to sucrose alone at the end of the experiment were included in the analysis. Typically the CS duration was in the range of 3–5 s, the US duration equaled 3–5 s, and the CS-US overlap equaled 1–2 s. The occurrence of the proboscis extension during the CS not overlapping with the US was documented in a binary form as the CR. For conditioning trial *t* we denote the absence or presence of the CR with *x*_*t*_ = 0 or *x*_*t*_ = 1, respectively. Table 1 provides an overview over the experimental data analyzed in this study. Details for each dataset are provided in the following.

### Absolute conditioning data (Datasets 1–12)

Datasets 1–12 comprise data on olfactory classical conditioning with a single CS, referred to as absolute conditioning. Animals in data sets 1, 10–12 were tested twice for memory retention (see Table 1). For consistency we did not analyzed the first test. CS duration, US duration, and CS-US overlap equaled 5, 4, and 2 s, respectively.

### Trace and delay conditioning data (Dataset 13–15)

Dataset 13 comprises data on trace conditioning (compare with Figure 2Aii (trace) in Szyszka et al., 2011). CS duration and US duration equaled 0.5 and 3 s, respectively. The CS and the US did not overlap. The gap between CS offset and US onset was 4.5 s. Dataset 14 comprises data on delay conditioning (compare with Figure 2Aii (delay) in Szyszka et al., 2011). CS duration, US duration, and CS-US overlap equaled 6, 3, and 1 s, respectively. Dataset 15 comprises data in which the time difference between the onset of the CS and the US was systematically varied in 8 subgroups of animals (compare with Figure 2Bii in Szyszka et al., 2011, CS-US onset differences equaled −6, 0, 1, 2, 3, 6, 10, and 15 s). CS durations and US durations equaled 0.5 and 3 s, respectively.

### Olfactory and tactile conditioning data (Dataset 16, 17)

Dataset 16 and 17 comprise data on olfactory and tactile conditioning (compare with Table 1 in Scheiner et al., 2001a). We did not differentiate between honeybees from low and high genetic strains. As was shown in the original study (Scheiner et al., 2001a), animals from low and high genetic strains did not differ in learning performance if they had the same GRS. For tactile conditioning small rectangular copper plates with vertical grooves were used as the CS (for details see Erber et al., 1998; Scheiner et al., 1999, 2001a) and sucrose was used as US and reward. The US was the same in olfactory and tactile conditioning. Prior to the conditioning session individuals were tested for their responsiveness to sucrose by touching their antennae with 9 different sucrose concentrations [1, 1.6, 2.5, 4, 6.3, 10, 16, 25, and 40% (w/v)]. Between the sucrose stimulations, antennae were touched with water to test for sensitization effects. The inter-trial-interval was 2 min to avoid intrinsic sensitization. For each animal the total number of proboscis responses to the first water and the nine sucrose stimulations was counted. This sum is referred to as the gustatory response score (GRS) of a bee (Scheiner et al., 2004). In the conditioning session, animals were trained by 10 pairings of CS (citral, 2 μ l added to airstream for 3 s before onset of the sucrose stimulation) and US (0.2 μ l 30% sucrose solution) at an inter-trial-interval of 5 min. Twenty-four hours after conditioning, bees were exposed to five unreinforced CS. In the present analysis we only included the first CS-only trial as a memory retention test and disregarded all subsequent trials. In each trial, the CS was given 3 s before the onset of the US at the antennae, which was followed by a proboscis stimulation with sucrose. The CS-US overlap was 1 s and the US duration at the proboscis was 1 s. It should be noted that for dataset 16 and 17 equal proportions of animals from different ranges of GRSs were collected, hence the datasets do not comprise a random sample of animals. Consequently, these two datasets are not considered when calculating the mean of the parameters *CR*_*stability*_ and *t*_*firstCR*_ over different datasets.

### Massed and spaced conditioning data (Datasets 18, 19)

Datasets 18 and 19 comprise animals from massed and spaced training conditions (Menzel et al., 2001). Under massed training conditions inter-trial-intervals equaled 30 s, while under spaced training conditions inter-trial-intervals equaled 15 min. We included all animals that survived the conditioning session in our analysis (group sizes differ from Menzel et al., 2001). CS duration, US duration, and CS-US overlap equaled 4, 3, and 1 s, respectively.

### Differential conditioning data (Dataset 20)

Dataset 20 comprises data on differential classical conditioning where two groups of animals were conditioned by 6 rewarded (CS+) and 6 unrewarded (CS−) odor presentations. The first group received 1-hexanal and 1-octanol as CS+ and CS− respectively, while in the second group the odor reward contingencies were reversed. Conditioning started with a CS+ trial and then alternated between CS− and CS+. The inter-trial-interval between identical stimuli equaled 14 min. Animals were tested for memory retention and discrimination at 1 and 24 h. CS+ (CS−) duration, US duration, and CS-US overlap equaled 5, 4, and 2 s, respectively.

### Experiment 1 (Datasets 21, 22)

Experiment 1 was performed in the summer of 2011 with honeybee foragers (*Apis mellifera*) from outdoor hives. Bees were caught, fed until satiation and starved overnight. One hour before conditioning bees which showed a proboscis extension reflex to a 1 M sucrose reward were selected for the experiment. Each bee was put into a conditioning chamber where she stayed throughout the entire experiment (training, resting, and testing) to reduce contextual changes. During classical conditioning bees were trained to associate either 1-hexanol or 1-nonanol (CS) with a 1 M sucrose reward (US). The odorants were diluted 1:100 in mineral oil (Sigma-Aldrich, Deisenhofen, Germany), and were presented as 4-s long stimuli with a custom-made olfactometer (Szyszka et al., 2011). US duration and CS-US overlap equaled 3 and 2 s, respectively.

Experiment 1 was designed to obtain bees that fall into one of the following four subgroups: 0111, 01, 001, 0001. The binary notation equals the sequence of CRs during the conditioning session, referred to as the CR-history. The leftmost number equals the CR in the first conditioning trial, while the rightmost number equals the CR in the last conditioning trial. To obtain these subgroups at comparable sample sizes we chose the following experimental protocol: In each experimental run, 16 bees were conditioned in parallel. Out of these bees, four animals were conditioned four times without interfering with the conditioning process (dataset 21). Another eight bees were conditioned until the first CS-evoked proboscis extension, yielding the CR histories 01, 001, 0001, and 0000. The remaining 4 bees were conditioned 4 times in case they showed a proboscis extension in the second trial or until the first proboscis extension otherwise. The 12 animals per plate conditioned by the latter two protocols are referred to as dataset 22.

Memory retention was tested 24 h after training. During the test, each bee was stimulated with the CS and a new odorant which in addition allowed the calculation of a discrimination index (*DI*, see below) by subtracting the response to a new odorant from the response to the CS (Biergans et al., 2012; Matsumoto et al., 2012). This procedure eliminates all non-associative effects of the conditioning procedure, such as sensitization or pseudo conditioning, which would also increase animals' responsiveness (Tully, 1984). 1-Hexanol and 1-nonanol were equally often used as CS and new odorant. For each behavioral response we also recorded its duration to capture possible differences in memory strength (Smith and Menzel, 1989). Response duration was measured as time (in 1-s intervals) between the beginning of the horizontal proboscis extension until its first retraction below the horizontal position. In case of no response, no duration value was incorporated. The inter-trial interval was 10 min both in training and in the test. The discrimination index *DI* was computed as

(1) $$DI=\frac{1}{N}\sum_{i\;=\;1}^{N}x_{CS}^{i}-x_{new}^{i}$$

where *x*^*i*^ denotes the CR of animal *i* to the presentation of the CS and new odorant, and *N* equals the number of animals in a given subgroup defined by the CR history. The discrimination index based on the CR duration was computed as

(2) $$DI_{dur}=\frac{1}{N}\sum_{i\;=\;1}^{N}\frac{d_{CS}^{i}-d_{new}^{i}}{max\left(d_{CS}^{i},d_{new}^{i}\right)}$$

where *d*^*i*^ denotes the duration of the proboscis extension of animal *i* to the CS and new odorant. Differences between durations of proboscis extensions were normalized individually by the maximum duration of animal *i* to either stimuli.

### Experiment 2 (Datasets 23, 24)

Experiment 2 was performed in late autumn/winter 2011 with honeybee foragers (*Apis mellifera*) from indoor hives. Bees were treated as in experiment 1. Bees either experienced two-trial conditioning (dataset 23) or single-trial conditioning plus a CS presentation without sugar reward 10 min after conditioning (dataset 24). This yielded the CR-history subgroups 01 and 0(1). The bracket notation indicates the CR in the CS-only trial. Memory retention and discriminatory power was measured as described in Experiment 1, and 1-hexanol and 1-nonanol were used equally often as CS and new odorant. Animals of data sets 23 and 24 were conditioned in parallel.

### Experiment 3 (Dataset 25)

Experiment 3 was performed from April to July 2014 with honeybee foragers (*Apis mellifera*) from outdoor hives. It was designed to investigate the translation-dependency of memory retention after single-trial and three-trial conditioning. Bees were treated as in experiment 1. We used the translation inhibitor emetine following the protocol described in Friedrich et al. (2004). Eighteen hours before conditioning, bees were fed until satiation. Thirty min before conditioning bees were injected with 1 μl emetine solution (10 mM in saline) or saline (in mM: 130 NaCl, 7 CaCl2, 6 KCl, 2 MgCl2, 160 sucrose, 25 glucose, 10 HEPES, pH 6.7, 500 mOsmol) into the flight muscle. Two charges of emetine were purchased from Sigma-Aldrich in April and in June for the experiments done from April to June and from June to July, respectively. The emetine solution was prepared immediately before injection. Three-trial conditioning was performed with a 10-min inter-trial interval. Each experimental run was done with 16 bees which were equally split into the four experimental groups (single-trial or three-trial conditioning with emetine or saline injection). After single-trial conditioning, bees received a CS-only trial after 10 min. During the 24 h retention test we presented the CS and a novel odor to test for the odor specificity of the memory.

### Data analysis

**Figure 1:**
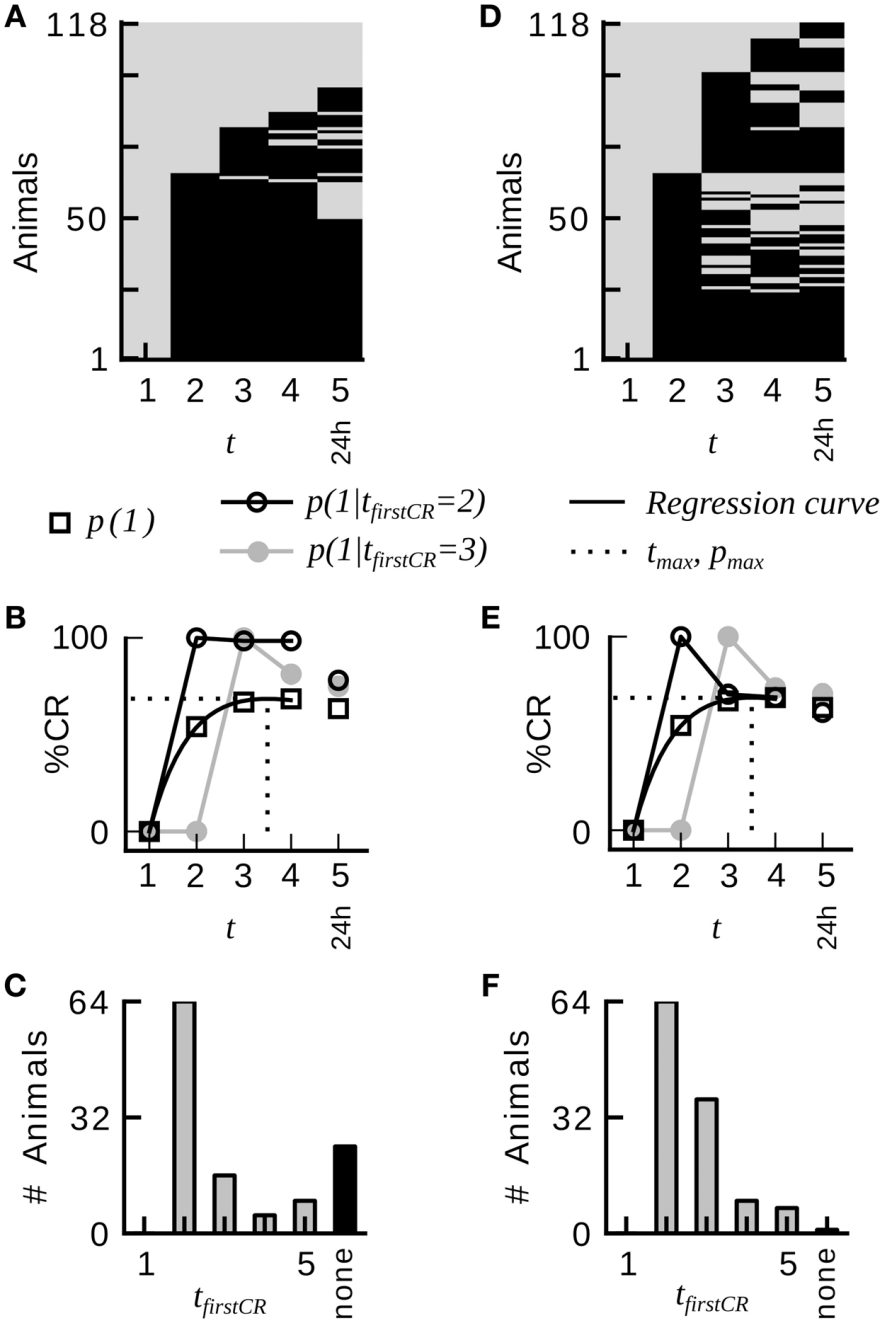
**Group-average CR probabilities do not adequately represent the CR probabilities in individual honeybees during classical conditioning of the proboscis extension response**. **(A)** Binary conditioned response matrix from a typical dataset consisting of four conditioning trials and one memory retention test at 24 h (dataset 21). A gray entry indicates no CR, a black entry indicates a CR. Animals have been sorted according to their first CR and the number of consecutive CRs. **(B)** Average CR probability *p*(1) and conditional CR probabilities *p*(*x*_*t*_ = 1 | *t*_*firstCR*_ = 2) and *p*(*x*_*t*_ = 1 | *t*_*firstCR*_ = 3). Once animals have initiated their first response, they remain responding in subsequent trials with high probability. The dotted line indicates the time point in trial time *t*_*max*_ at which the regression curve (Equation 3) on the average CR probabilities assumes its maximum. **(C)** Histogram of first responses. The largest proportion of animals starts to respond on the second trial. **(D)** Binary conditioned responses matrix of a hypothetical dataset, which was generated by randomly permuting the CRs of dataset 21 across animals for each trial separately. **(E,F)** Analog analysis to **(B,C)**. Group-average behavior represents individual behavior in the hypothetical dataset. Conditional probabilities do not reveal a serial dependency. The percentage of non-responders is drastically reduced.

An example raw dataset of binary CRs from absolute conditioning is depicted in Figure 1A. The data was analyzed by the following standard procedure: The notation *x*_*t*_ = 1 (*x*_*t*_ = 0) denotes the presence (absence) of the CR on trial *t*. The trial index *t* ranges from 1 to the maximum number of trials, including the memory test. The average CR probability equals the percentage of animals showing a CR in trial *t*. Average CR probabilities across trials were fitted by the equation

(3) $$p(CR)=a\left(1-e^{-b(t-1)}\right)+c(t-1)$$

where the three free parameters *a*, *b*, and *c* were estimated by least-squares minimization. The point in trial time at which the regression curve assumes its maximum *p*_*max*_ is denoted as *t*_*max*_ (Figure 1B). Animals in each dataset were divided into disjunctive subgroups defined by the trial *t*_*firstCR*_ at which animals showed their first CR (see Figure 1C for a histogram of first CRs). Animals that did not show a response in any of the trials constituted the subgroup of non-responders. For all occurring first CR indexes *j*, we computed the conditional probabilities *p*(*x*_*t*_ = 1 |*t*_*firstCR*_ = *j*) with *t* > *j*. Figure 1B exemplifies this analysis for *p*(*x*_*t*_ = 1 | *t*_*firstCR*_ = 2) and *p*(*x*_*t*_ = 1 | *t*_*firstCR*_ = 3). Taking the mean over all conditional probabilities *p*(*x*_*t*_ = 1 | *t*_*firstCR*_ = *j*) with *t* > *j* results in the CR stability of a subgroup defined by *t*_*firstCR*_ = *j*. Taking the weighted mean of the CR stabilities of all subgroups results in the overall CR stability of a given dataset. The CR stabilities of subgroups were weighted according to subgroup sizes. The CR stability is a measure of how constantly individuals of a given dataset responded once they had started to respond. From the definition follows that neither bees that do not show a CR in any of the trials (non-responders), nor animals that only respond in the last trial contribute to this parameter.

### The classical Rescorla–Wagner model

The Rescorla–Wagner (RW) model assumes that associative learning during classical conditioning is driven by prediction errors (Rescorla and Wagner, 1972; Sutton and Barto, 1990). At each conditioning trial *t* the animal experiences a prediction error (λ − *v*_*t*_) defined as the difference between the maximum associative strength λ supported by the US, and the associative strength *v*_*t*_ of the CS at the current trial. In the following we refer to the parameter λ ∈ [0, 1] as the US effectiveness. After each trial, the associative strength *v_*t*_* is updated according to the rule

(4) $$v_{t\;+\;1}=v_{t}+\alpha (\lambda -v_{t})$$

where α ∈ [0, 1] is the learning rate, defined in the original theory as the product of CS and US salience (Rescorla and Wagner, 1972). The update rule leads to a gradual strengthening of associative strength across conditioning trials (**Figure 7A**). Here we assume a linear mapping between associative strength and CR probability, hence the probability of animal *i* to show a CR on trial *t* equals *vt*, and the probability for not showing a CR equals 1 − *vt*. In Equation (4) the value *vt* denotes the associative strength at precisely the time of trial *t*, hence before the actual learning induced in this trial has become effective. This is analogous to the experimental situation in which the behavior observed in trial *t* is taken as a monitor of the associative strength induced in all previous trials. The two free parameters α and λ were estimated by minimizing the negative log-likelihood of the model on a given dataset by the L-BFGS-B algorithm for bound constrained optimization (Byrd et al., 1994; Zhu et al., 1997). The bounds for α and λ were set to [0, 1]. The starting value for α and λ were determined by a grid search on the range [0, 1] with a grid distance of 0.1.

### The Rescorla–Wagner model with heterogeneous learning performance *RW*^*P*(α, λ)^

In order to account for heterogeneous learning performance within a group of identically treated animals we employed the Resorla–Wagner model as follows: for each animal in a given dataset we computed the likelihood for observing its behavioral responses given different combinations of the learning parameters α and λ (**Figures 7C–F** depict four examples for animals emitting the CR sequences 01111, 01010, 00111, and 00000). In order to estimate the total probability distribution *P*(α, λ) over learning parameters in a given dataset we summed up the likelihoods of all individuals from that dataset. The total probability distribution was then normalized by the number of animals in the dataset. **Figure 7B** illustrates this total probability distribution *P*(α, λ) for dataset 21. Probability distributions were calculated at a grid distance of 0.1 for both learning parameters. We computed the eligibility of the classical and the extended Rescorla–Wagner model by a four-fold cross-validation algorithm. Data analyses were carried out in Python.

## Results

### The CR is stable within individual honeybees

**Figure 2:**
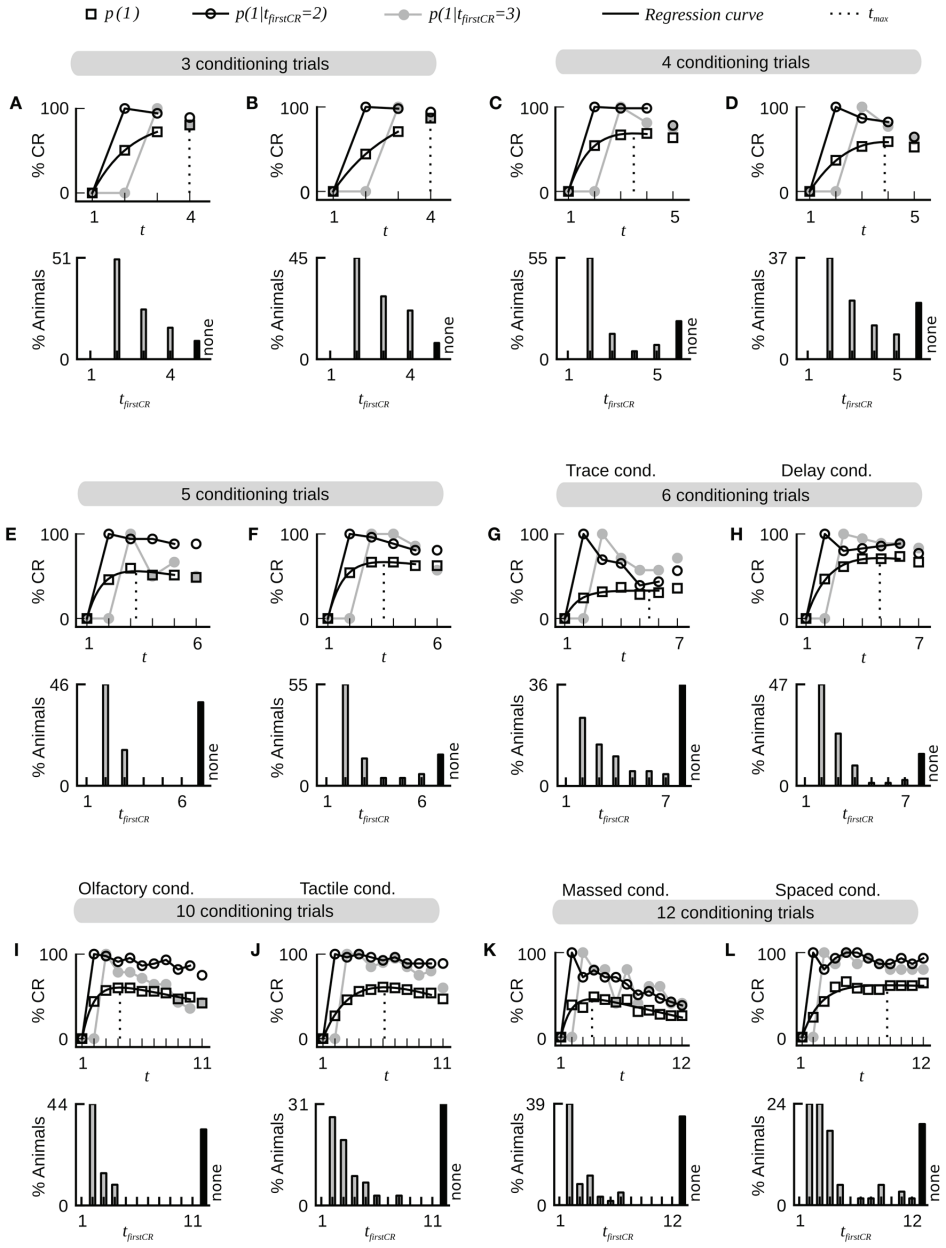
**Fast dynamics of associative learning during classical conditioning of the proboscis extension response**. For each data set the upper panel shows the average CR probabilities and the CR probabilities in two subgroups of animals that start to respond on the second (*t*_*firstCR*_ = 2) or third trial (*t*_*firstCR*_ = 3). The black line depicts a regression curve (Equation 3) on the average CR probabilities (open square symbols). The dotted line depicts the position *t*_*max*_ of the maximum of the regression curve in trial time. The lower panel displays the percentage of animals that showed their first CR in a given trial. Animals that did not show a CR in any of the trials are represented by the black bar (none). Across all data sets, the largest proportion of animals starts to respond after only a single conditioning trial. Once animals have responded for the first time they have a high probability to continue responding in subsequent trials. The percentage of non-responding animals varies across datasets. Bees which responded to the first CS before the CS-US pairing were excluded from the analysis. **(A)** Dataset 4. **(B)** Dataset 6. **(C)** Dataset 21. **(D)** Dataset 10. **(E)** Dataset 11. **(F)** Dataset 12. **(G)** Dataset 13: The CR stability is decreased under trace conditioning (4.5 s gap between CS offset and US onset). **(H)** Data set 14: Control group for dataset 13 (CS and US overlap by 0.5 s). **(I,J)** Datasets 16 and 17: The dynamics of olfactory associative learning resemble the dynamics of tactile associative learning. **(K)** Dataset 18: The stability of the CR is decreased under massed training conditions (inter-trial-interval equals 30 s). **(L)** Dataset 19: The stability of the CR is high under spaced training conditions (inter-trial-interval equals 15 min).

A typical dataset of binary behavioral responses (CR matrix) from absolute classical conditioning is depicted in Figure 1A, while Figures 1B,C exemplify the performed data analysis. We first asked how persistently individuals kept responding during conditioning once they had shown their first response. Quantifying this behavioral feature by a parameter termed CR stability (see Sections Materials and Methods, Data analysis), we found that the mean CR stability across all datasets with standard training conditions equaled (86.4 ± 6.5)% (mean ± SD throughout the paper) (datasets 1–12, 14, 19, 21). Hence once individuals had elicited their first response, they kept responding in all subsequent trials with a high probability (Figures 1, 2; Table 2). Dissecting the CR stability further we found that animals responding early had a higher CR stability than animals responding later: The CR stability of animals showing their first CR on the second trial equaled (89.7 ± 5.4)% (datasets 1–12, 14, 19, 21), for animals showing their first CR on the third trial it equaled (83.4 ± 10.3)% (datasets 1–12, 14, 19, 21), and for animals showing their first CR on the fourth trial it equaled (66.3 ± 14.2)% (datasets with more than four trials: 10, 12, 14, 19, 21) [One-Way ANOVA with trial as factor, *F*_(2, 32)_ = 10.83, *p* < 0.001]. In individual datasets this overall decrease was seen in 11 out of 15 datasets between the second and third trial, and in four out of five datasets between the third and fourth trial.

**Table 2:** **Summary of estimated parameters describing the dynamics of associative learning in individuals**.

**Dataset**	***N***	***CR*_***stability***_ (*%*)**	***CR*_***stability***_(*t*_***firstCR***_ = *2*) (*%*)**	***Mean*(*t*_***firstCR***_) (*%*)**	***N*_***non-responders***_/*N* (*%*)**
1	64	82.5 (40)	80.8 (26)	2.8	17.2
2	58	95.6 (45)	94.3 (35)	2.5	8.6
3	87	91.7 (60)	87.9 (33)	2.8	8
4	517	87.8 (389)	91.8 (261)	2.6	9.1
5	98	90.3 (77)	91.7 (54)	2.6	7.1
6	113	92.6 (81)	96.0 (50)	2.8	7.1
7	92	87.9 (66)	90.0 (40)	2.7	9.8
8	85	90.1 (71)	93.9 (49)	2.5	4.7
9	94	94.2 (69)	97.5 (40)	2.7	8.5
10	122	70.9 (86)	77.8 (45)	2.9	20.5
11	37	81.9 (23)	91.2 (17)	2.3	37.8
12	48	81.5 (37)	86.5 (26)	2.7	16.7
13	95	52.2 (57)	54.8 (23)	3.5	35.8
14	75	83.1 (62)	82.9 (35)	2.8	14.7
15, 1 s	35	76.5 (26)	80.0 (6)	3.7	20
15, 2 s	34	65.0 (17)	70.0 (4)	3.8	44.1
15, 3 s	34	62.9 (19)	80.0 (4)	3.8	44.1
15, 6 s	42	71.8 (10)	60.0 (1)	3.6	73.8
15, 10 s	34	37.1 (7)	80.0 (2)	4	76.5
15, 16 s	31	34.5 (10)	31.4 (7)	3	64.5
16, GRS 10	33	91.8 (31)	95.6 (25)	2.2	6.1
16, GRS 8–9	25	69.1 (17)	80.8 (11)	2.5	32
16, GRS 5–7	22	73.8 (16)	100.0 (5)	3	27.3
16, GRS 2–4	20	37.0 (3)	37.0 (3)	2	85
17, GRS 10	42	86.4 (40)	95.6 (20)	3	4.8
17, GRS 8–9	29	77.6 (21)	82.2 (5)	3.5	27.6
17, GRS 5–7	16	73.4 (8)	94.4 (2)	4.2	50
17, GRS 2–4	13	– (0)	– (0)	0	100
18	63	52.6 (42)	58.3 (24)	3	33.3
19	64	77.9 (51)	91.3 (15)	4	18.8
20, CS+	98	93.7 (87)	97.0 (61)	2.8	6.1
21	118	88.4 (85)	91.7 (64)	2.6	20.3

N, Number of animals in each dataset. The parameter CR_stability_ equals the mean probability to respond in subsequent trials, given that animals have started to respond in any of the trials. Numbers in brackets denote numbers of responding animals. The parameter CR_stability_(t_*firstCR*_ = 2) denotes the mean probability to respond in subsequent trials, given that animals have started to respond in the second conditioning trial. Numbers in brackets indicate the numbers of animals starting to respond in the second trial. Mean(t_*firstCR*_): Mean time-point in trial time at which animals display their first CR, given that animals respond in any of the trials. *N*_*non-responders*_/*N*: Proportion of animals that do not respond in any of the trials. For dataset 15 (trace conditioning) parameters were computed independently for different delays between CS and US onset as indicated. For datasets 16 and 17 parameters were computed for 4 subgroups defined by gustatory responsiveness scores (GRS). For dataset 20 parameters were computed for CS+ trials only.

### 54% of the animals start to respond after a single conditioning trial

Next we analyzed at which trial individuals typically showed their first CR. Histograms of first CRs in trial time are displayed in Figure 1C and in the lower panels of Figure 2. We found that (54.1 ± 11.4)% of the animals which showed at least one response in any of the trials started to respond in the second trial, i.e., after having experienced a single CS-US pairing (datasets 1–12, 14, 19, 21). By the third trial (80.6 ± 8.7)% of all responding animals had started to respond, and by the fourth trial (95.9 ± 6.4)% had started to respond. On average, the first CR was shown after 2.8 ± 0.4 trials (datasets 1–12, 14, 19, 21, Table 2). First CR histograms of datasets with many conditioning trials (5–12) furthermore imply that there is a population of animals that do not start to respond not even under prolonged training (Figures 2E–L, black histogram bars denote non-responders). The average percentage of non-responders equaled (21.5 ± 7.6)% [datasets 10, 12, 14, 19, 21, only datasets in which the maximum of the regression curve (Equation 3) was reached during conditioning were considered].

### Animals that responded already before training share the same learning dynamics as animals that started responding during training

**Figure 3:**
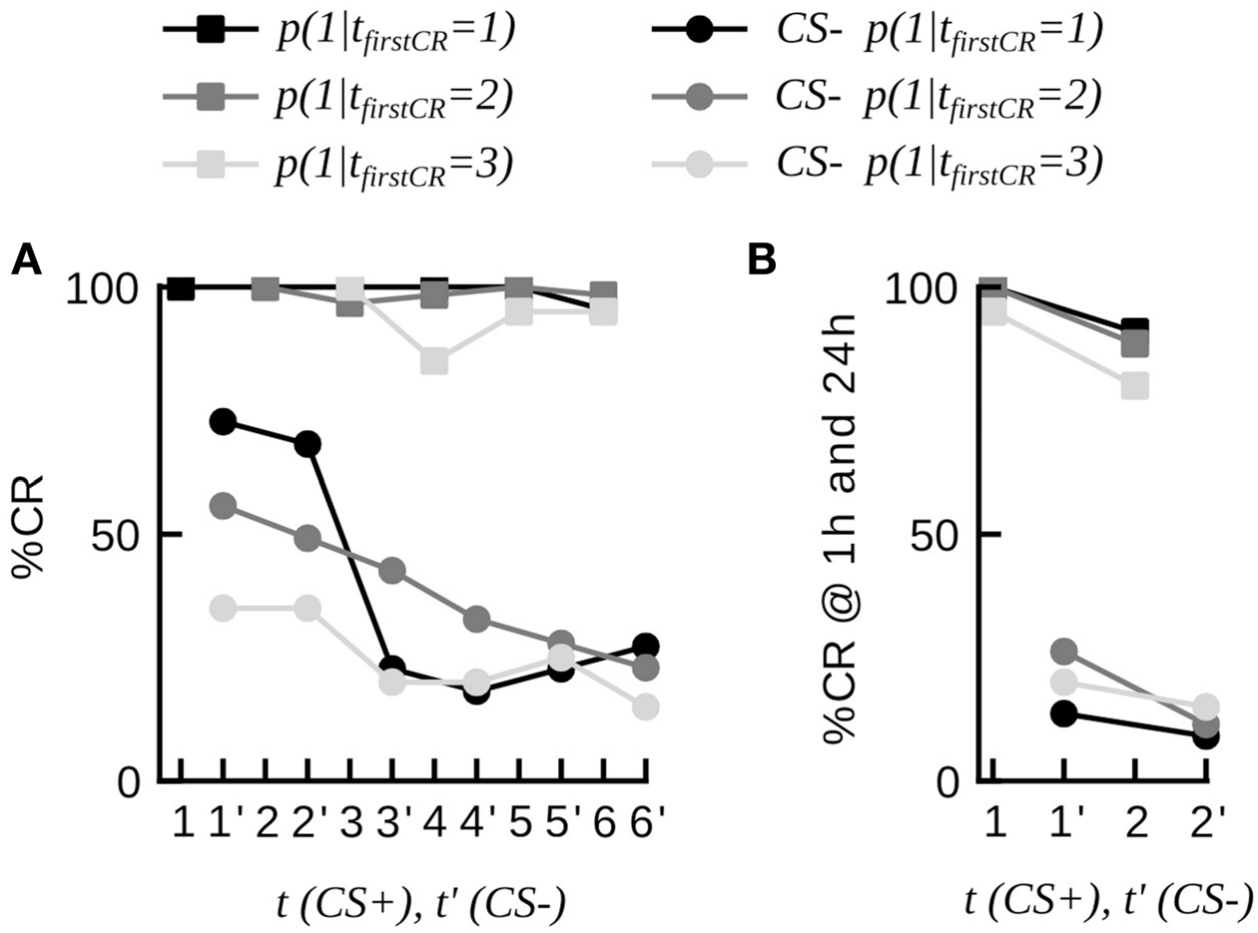
**Dynamics of discriminative learning during differential conditioning (data set 20). (A)** CR probabilities to the CS+ and the CS− during the conditioning phase in three subgroups of animals, defined by their first response trial to the CS+ (*t*_*firstCR*_ = 1, 2, 3). Once animals have started to respond to the CS+, they have a high probability to continue responding to the CS+ in consecutive trials (curves with square markers). Animals responding to the CS+ (*t*_*firstCR*_ = 1, 2, 3) show high CR probabilities to the CS− in the first conditioning trials, and low CR probabilities to the CS− at the end of the conditioning phase (curves with round markers). CS− trials are indicted by an apostrophe. **(B)** CR probabilities to the CS+ and the CS− of the three subgroups at 1 and 24 h.

It has often been debated among experimenters whether or not honeybees responding to the CS in the very first conditioning trial should be in- or excluded from the experimental sample (Matsumoto et al., 2012). In some of the raw datasets animals that extended their proboscis to the first CS presentations were not excluded by the experimenter (total *N* = 96, datasets 3–12, 14). For consistency we have so far not included these animals in our analysis. We asked if these spontaneous responders (*t*_*firstCR*_ = 1) reliably responded to the CS in subsequent trials. We found that this was the case by computing the CR stability of these animals, which equaled (85.0 ± 12.8)%. We furthermore found that spontaneous responders discriminated well between the CS+ and the CS– during differential conditioning as well as in a subsequent memory test (21 animals of dataset 20, Figure 3). This suggests, that spontaneous responders share the same learning dynamics as animals responding later (e.g., on the second trial), and hence do not have to be removed from the training group in future experiments.

### The relationship between individual dynamics and group-average behavioral dynamics

Most animals showed early and stable CRs during the training phase. However, a portion of animals did not respond in any of the trials. How can these learning dynamics in individuals be reconciled with the learning dynamics apparent at the population level, often referred to as the “learning curve” or “acquisition function?” We described the learning dynamics at the population level by two parameters: The maximum *p*_*max*_ of the regression curve (Equation 3) on the average CR probabilities, typically referred to as the asymptote of learning, and the position of this maximum *t*_*max*_ in trial time (Section Materials and Methods, this analysis is exemplified in Figure 1B). We found that *t*_*max*_ coincided with the cessation of first responding across the population. By this time-point, (91.3 ± 5.2)% of the animals that showed at least one CR in any of the trials had started to respond (datasets 10, 12, 14, 19, 21, only datasets in which the maximum was reached during conditioning were considered). The value of *p*_*max*_ reflected two behavioral characteristics at the level of individuals, the proportion of non-responding animals *N*_*non-responders*_/*N* and the CR stability. We found that the following rule of thumb

(5) $$p_{max}\approx CR_{stability}\left(1-N_{non\text{-}responders}/N\right)$$

holds for all datasets with only a small error of (0.8 ± 1.8)% (datasets 10–12, 14, 16, 19, 21). This relation illustrates that the parameter *p*_*max*_ does not represent a performance asymptote of individual learning. Instead it represents the percentage of responding animals in the population, modulated by their asymptotic response probability (CR stability).

The same finding applies to the memory retention test. The group-average CR probability in the retention test did not represent memory retention in individual honeybees. For animals that showed at least one CR in any of the conditioning trials the CR probability in the retention test equaled (72.0 ± 6.7)% (datasets 10, 11, 12, 14, 19^*^, 21. Only datasets in which the maximum *p*_*max*_ was assumed during conditioning were taken into account. Dataset 19^*^ consisted of a subgroup of animals from dataset 19 that survived until the retention test at 72 h). However, memory retention in animals that never responded during conditioning equaled only (24.2 ± 13.7)%.

### Multiple-trial and single-trial conditioning can produce indistinguishable 24 h memory retention

How does the number of conditioning trials affect the stability or strength of the induced memory? The prevailing hypothesis states that three-trial conditioning induces a stable memory, expressed in a high CR probability 24 h after training, whereas single-trial conditioning induces a weaker memory with a low 24 h retention probability (Menzel, 1990, Figure 9.8; Müller, 2012, Figure 1; Menzel, 2012, Figure 2).We asked whether the commonly found difference between group-average retention probability after three-trial and single-trial conditioning indeed reflects enhanced memory retention in individuals after more training, or whether it primarily reflects an increased proportion of learners. A single conditioning trial may already be sufficient for a subgroup of animals to learn the CS-US association which is expressed by a stable CR. More conditioning trials may only further increase the proportion of learners, but they may not have any effect on animals that already responded after the first trial.

**Figure 4:**
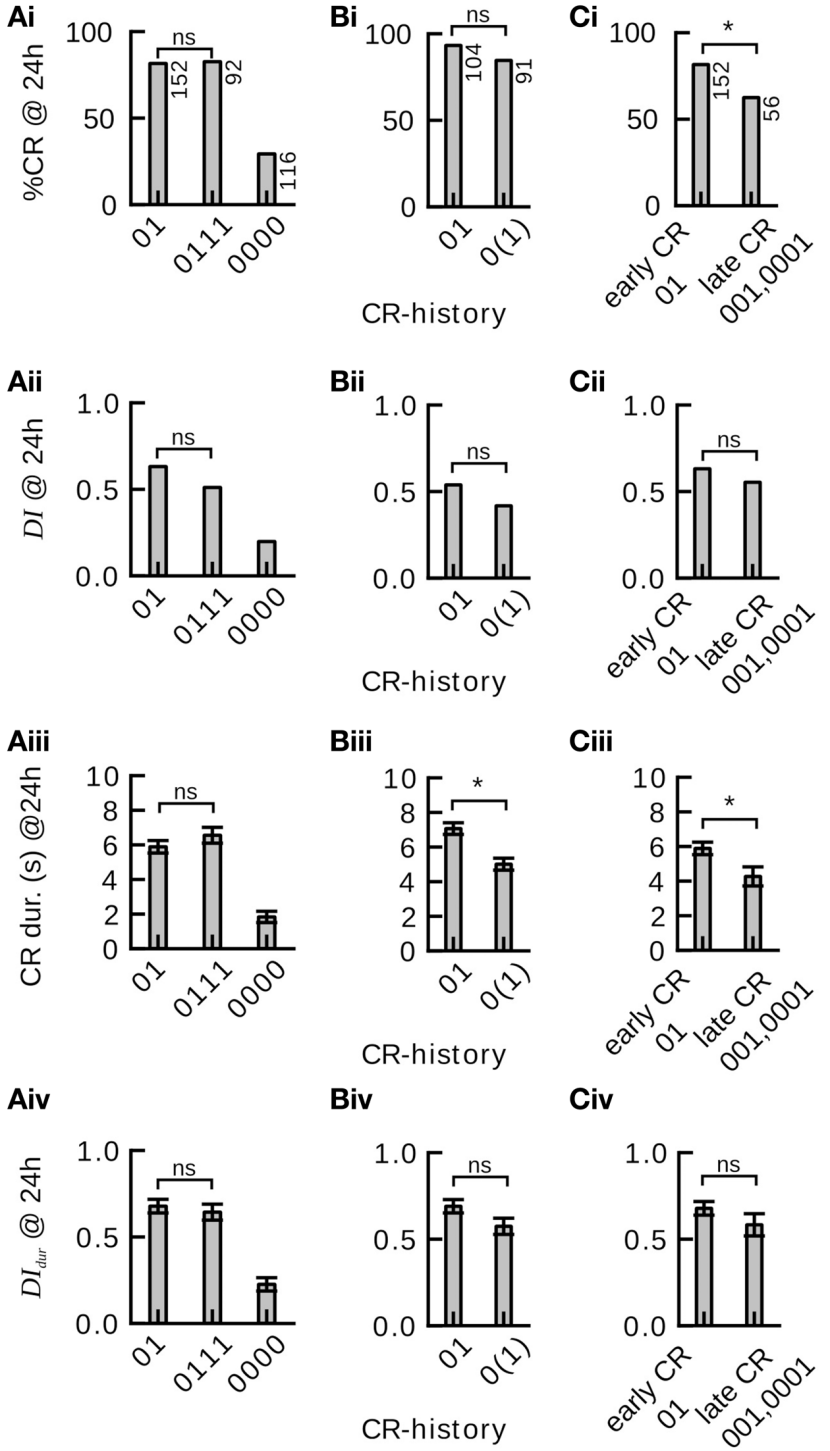
**Effect of single-trial and multiple-trial conditioning on 24 h memory retention and discriminatory power under examination of individual CR histories during conditioning**. ^*^P < 0.05. **(Ai)** CR probability to the trained odor in subgroups 01, 0111, and 0000 of Experiment 1. Memory retention after four-trial and two-trial conditioning is not significantly different (0111 vs. 01 subgroup, χ^2^ = 0.000960 with 1 degrees of freedom, *P* = 0.975). Animals that did never respond during four-trial conditioning showed poor memory retention. **(Aii)** Discrimination index (DI) in subgroups 01, 0111, and 0000. Discriminatory power of the memory after four-trial conditioning and two-trial conditioning is not significantly different (0111 vs. 01 subgroup, Mann–Whitney Rank Sum Test, *T* = 10379.000, *P* = 0.095). Animals that did not respond during four-trial conditioning show poor memory discrimination. **(Aiii)** Duration of the proboscis extension to the trained odor in subgroups 01, 0111, and 0000. The CR duration is not significantly different after four-trial and two-trial conditioning (0111 vs. 01 subgroup, Mann–Whitney Rank Sum Test, *T* = 11901.500, *P* = 0.238). **(Aiv)** Discrimination Index computed on CR duration (*DI*_*dur*_) in subgroups 01, 0111, and 0000. The CR duration does not reveal significant differences in memory discrimination after four-trial and two-trial conditioning (0111 vs. 01 subgroup, Mann–Whitney Rank Sum Test, *T* = 10652.500, *P* = 0.248). **(Bi)** Memory retention after two-trial and single-trial conditioning is not significantly different (Experiment 2, 01 vs. 0(1) subgroup, χ^2^ = 2.935 with 1 degrees of freedom, *P* = 0.087). **(Bii)** The discrimination index after two-trial and single-trial conditioning is not significantly different (01 vs. 0(1) subgroup, Mann–Whitney Rank Sum Test, *T* = 8346, *P* = 0.146). **(Biii)** The duration of the proboscis extension response to the trained odor is significantly longer after two-trial than after single-trial conditioning (01 vs. 0(1) subgroup, Mann–Whitney Rank Sum Test, *T* = 7265.5, *P* ≤ 0.001). **(Biv)** The duration of the proboscis extension response did not reveal significant differences in discriminatory power after two-trial and single-trial conditioning (01 vs. 0(1) subgroup, Mann–Whitney Rank Sum Test, *T* = 8256.5, *P* = 0.093). **(Ci)** Animals that started to respond early during conditioning showed significantly more memory retention than animals that started to respond later during conditioning [Experiment 1, 01 vs. (001, 0001) subgroup, χ^2^ = 7.246 with 1 degrees of freedom, *P* = 0.007]. **(Cii)** Early and late responders do not significantly differ in memory discrimination (Mann–Whitney Rank Sum Test, *T* = 5489, *P* = 0.346). **(Ciii)** The duration of the proboscis extension response to the trained odor is significantly longer in early than in late responders (Mann–Whitney Rank Sum Test, *T* = 4925.5, *P* = 0.016). **(Civ)** The duration of the proboscis extension response does not reveal significant differences in memory discrimination between early and late responders (Mann–Whitney Rank Sum Test, *T* = 5428, *P* = 0.271).

In order to study the effect of single-trial and multiple-trial conditioning on 24 h memory retention and discriminatory power in individuals we carried out two experiments (Experiments 1 and 2, see Section Materials and Methods). We found that memory retention after four-trial conditioning in individuals with a CR-history of 0111 did not significantly differ from memory retention after two-trial conditioning (CR-history 01) (Figure 4Ai). (The CR-history denotes the sequence of CRs during conditioning with the symbols 0 (no response) and 1 (response).The leftmost symbol represents the outcome of the first conditioning trial and the rightmost symbol represents the outcome of the last conditioning trial.) In addition we found that memory retention after two-trial conditioning (CR-history 01) did not significantly differ from memory retention after single-trial conditioning (CR-history 0(1)) (Figures 4Bi,Bii). A CR-history of 0(1) denotes animals that experienced one CS-US pairing in the first trial, and extended their proboscis to an unrewarded CS in the second trial of the training phase.

We obtained the same result when looking at the discriminatory power of the induced 24 h memories (Figure 4Bii): The discrimination index (Equation 1) did not differ significantly between four-trial conditioning (CR-history 0111) and two-trial conditioning (CR-history 01), nor between two-trial (CR-history 01) and single-trial conditioning (CR-history 0(1)). Hence, for honeybees that responded after the first conditioning trial a single CS-US pairing was sufficient to induce a stable and odor-specific 24 h memory.

We also analyzed graded measures for 24 h memory retention and discrimination based on proboscis extension durations. These measures overall confirmed our previous results (Figures 4Aiii,Aiv,Biv). However, we found a significantly shorter proboscis extension duration to the CS after single-trial conditioning than after two-trial conditioning (Figure 4Biii).

**Figure 5:**
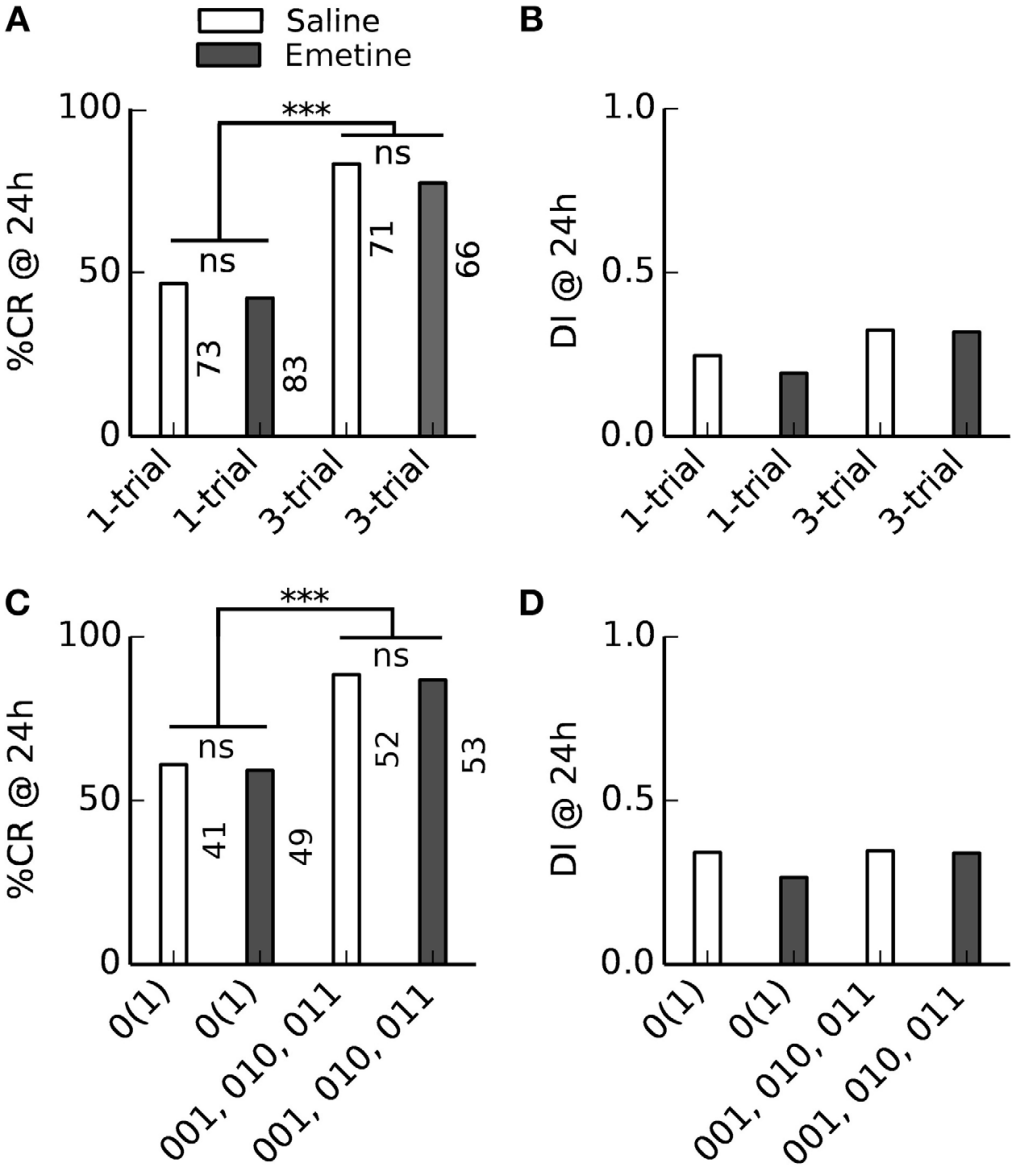
**Injection of the translation blocker emetine before conditioning had no effect on 24 h memory retention**. Bees received a saline or emetine injection 30 min before single- or three-trial conditioning. Memory retention was tested 24 h after conditioning. ^***^P < 0.001. **(A,B)** Averaged performance of all bees; **(C,D)** averaged performance of bees which showed a conditioned response (CR) during training. **(A)** The percentage of bees which showed a CR in the 24 h memory retention test differed between single-trial and three-trial conditioning [*F*_(1, 289)_ = 45, *p* < 0.001; Two-Way ANOVA] but not between emetine- and saline-injected bees [*F*_(1, 289)_ = 0.9, *p* = 0.34]. **(B)** The discrimination index (DI) of the 24 h memory retention test neither differed between single- and three-trial conditioning [*F*_(1, 289)_ = 3.3, *p* = 0.07] nor between emetine- and saline-injected bees [*F*_(1, 289)_ = 0.2, *p* = 0.66]. **(C)** The percentage of bees which showed a CR in the 24 h memory retention test differed between single- and three-trial conditioning [*F*_(1, 191)_ = 21.2, *p* < 0.001] but not between emetine- and saline-injected bees [*F*_(1, 191)_ = 0.1, *p* = 0.8]. **(D)** The DI of the 24 h memory retention test neither differed between single- and three-trial conditioning [*F*_(1, 191)_ = 0.2, *p* = 0.7] nor between emetine- and saline-injected bees [*F*_(1, 191)_ = 0.2, *p* = 0.6].

The indistinguishable 24 h memory retention after single-trial and three-trial conditioning conflicts with the pharmacological difference between single-trial and multiple-trial induced 24 h memories: Multiple-trial induced 24 h memory can be impaired when the translation inhibitor emetine is injected 30 min before conditioning (Friedrich et al., 2004; Stollhoff et al., 2005), while single-trial induced 24 h memory was not impaired (Friedrich et al., 2004). The indistinguishable 24 h memory retention after single-trial and three-trial conditioning which we found in learners could have two reasons: (1) In learners (bees which acquired a stable CR during conditioning), single-trial and multiple-trial induced 24 h memories are equally translation-dependent, or (2) under our experimental conditions single-trial and multiple-trial induced 24 h memories are not translation-dependent at all. To distinguish between these alternative explanations we compared the 24 h memory retention after single-trial and three-trial conditioning in bees which received either an injection of emetine or saline 30 min before conditioning (Figure 5). We found no difference in the retention or discriminatory power of the 24 h memory between saline- and emetine-injected bees. However, in contrast to Experiments 1 and 2 (Figure 4), 24 h memory retention was higher after more training trials in responding animals [compare (010, 001, 011) bees vs. 0(1) bees in Figure 5C], but there was no difference in discriminatory power (Figure 5D).

### Honeybees responding early show higher 24 h memory retention than those responding later

We asked whether the time point during conditioning at which individual honeybees showed their first CR (*t*_*firstCR*_) would have any effect on 24 h memory retention and discriminatory power. In particular we wanted to compare animals that showed the CR only once, while having experienced a different number of training trials. We designed the experiment such that animals did not receive further training after their first CR during conditioning (Experiment 1, Section Materials and Methods). Both in binary and graded measures we found that animals responding early (CR-history 01) showed higher memory retention than those responding later (CR-history 001 and 0001) (Figures 4Ci,Ciii), confirming the trend seen in our previous data analysis. However, when looking at the discrimination index, we found no significant differences between memory's discriminatory power in animals responding early and those responding later (Figures 4Cii,Civ). The discriminatory power captures the pure associative effect of the conditioning procedure, devoid of non-associative effects such as sensitization (Tully, 1984). Together, these results indicate that early responders have a higher general response probability, while the truly associative component of learning was at equal levels in early and late responders.

### The effect of different training conditions on learning parameters of individuals

We further asked how individual learning dynamics were affected by altered training conditions such as in trace conditioning, conditioning with massed training trials and differential conditioning.

In trace conditioning animals experienced a 4.5-s long stimulus-free gap between the CS and the US (dataset 13), whereas animals in the control group (delay conditioning) experienced a 1-s overlap between the CS and the US (dataset 14). At the population level, this resulted in a significantly lowered response probability at the end of the conditioning phase during trace conditioning as compared to delay conditioning (Chi-square test, *p* < 0.001). Accordingly the maximum of the regression curve *p*_*max*_ was lowered in trace conditioning as compared to delay conditioning (Figures 2G,H). We asked whether in this more difficult learning task the decrease was observed because individuals responded with lower probability to the CS, because fewer animals started to respond at all, or because of a combination of both factors. We found that both a significantly decreased CR stability and a significant increase in the number of non-responders were responsible for the decrease of *p*_*max*_ (Table 2, dataset 13, 14, Chi-square tests, *p*_*CR stability*_ < 0.001, *p*_*number of non-responders*_ < 0.01).The same trend was observable in six subgroups of dataset 15, in which the delay between the CS and US onset was systematically varied between 1 and 15 s (Table 2). However, this trend was not testable due to small sample sizes.

Comparing massed and spaced training conditions, we observed that massed trials at an inter-trial interval of 30 s (dataset 18, Figure 2K) resulted in a significant decrease of the response probability in the last conditioning trial as compared to the control group with spaced trials at an inter-trial interval of 15 min (dataset 19, Figure 2L, Chi-square, *p* < 0.001), and accordingly the asymptote *p*_*max*_ was lowered in massed conditioning as compared to spaced conditioning. We found that this decrease resulted from a significant decrease of CR stability in individuals (Chi-square, *p* < 0.001), while the percentage of non-responding animals was not significantly increased (Chi-square, *p* = 0.095).Under massed conditioning trials animals responding early (*t*_*firstCR*_ = 2, 3) initially had a high CR probability in subsequent trials but later on their CR probabilities decayed, which may be due to US habituation, inhibitory learning or satiation (Menzel et al., 2001).

Analyzing data from differential conditioning we found that the alternating presentation of unrewarded stimuli (CS−) during conditioning had no effect on the rapid and stable acquisition of a CR to the CS+ (dataset 20 Table 2, Figure 3).

### Sucrose responsiveness correlates with learning performance

**Figure 6:**
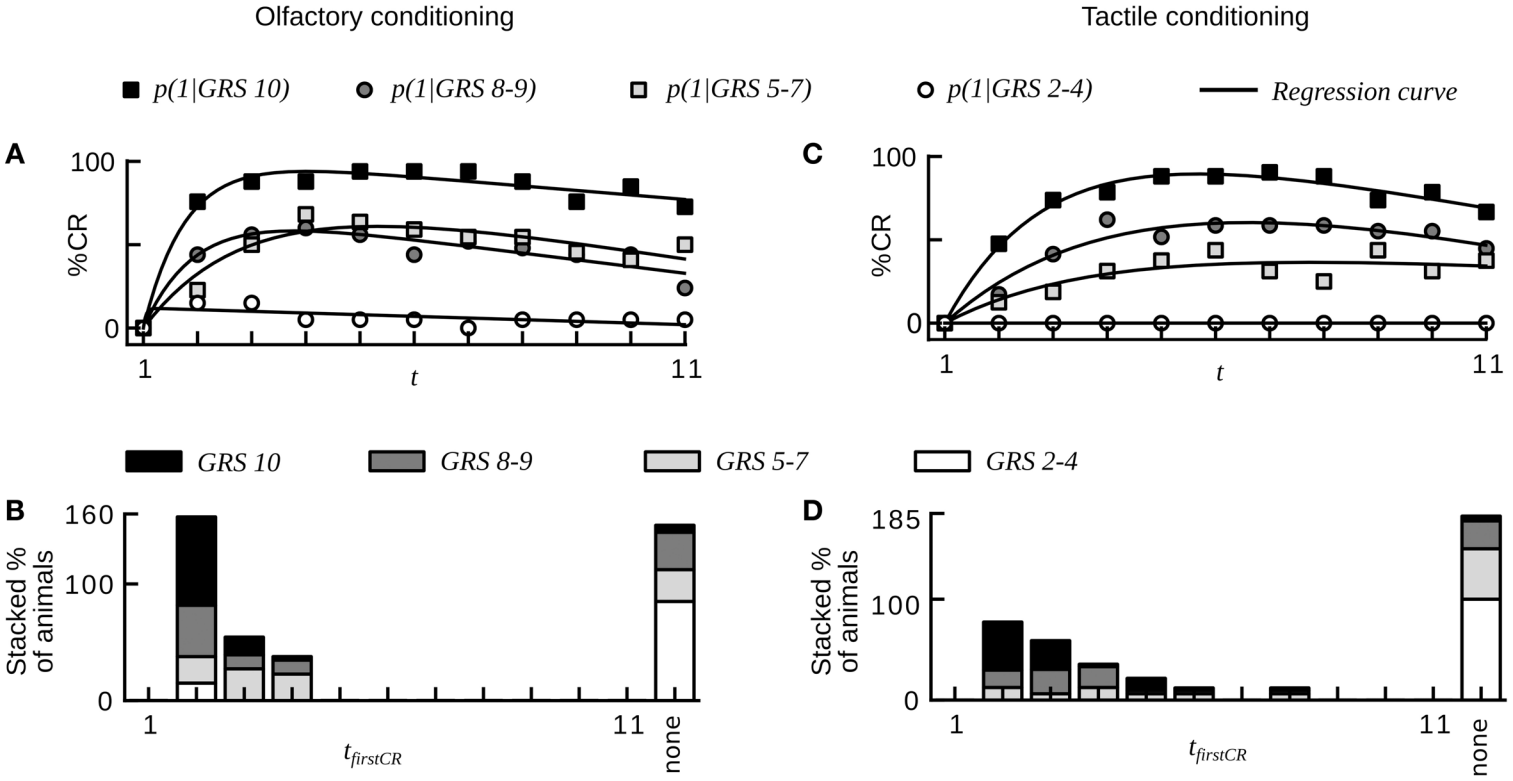
**Sucrose responsiveness correlates with learning performance in olfactory and tactile classical conditioning (compare to Figures 1, 2 in Scheiner et al., 2001a)**. **(A)** Average CR probabilities in four subgroups of animals from olfactory conditioning (dataset 16). Animals were divided into subgroups on the basis of individual gustatory response scores (GRS). Small numbers indicate low responsiveness to sucrose. Animals with high responsiveness to sucrose reach higher plateaus in CR probability than animals with low responsiveness to sucrose. **(B)** Histogram showing the percentage of animals for each subgroup that start to respond in a given trial. Animals showing at least one CR in any of the trials start to respond early during conditioning. Most non-responders have a low responsiveness to sucrose. **(C)** Average CR probabilities in four subgroups of animals from tactile conditioning (dataset 17). **(D)** Histogram showing the percentage of animals for each subgroup that starts to respond in a given trial. The dynamics of tactile learning resemble the dynamics of olfactory learning.

Our analysis showed that equally treated honeybees varied substantially in their learning performances both during simple absolute conditioning and more difficult conditioning tasks such as trace and differential conditioning. What was the reason for these learning differences across individuals? Several studies demonstrated that the responsiveness to sucrose correlates with learning performance in individual harnessed honeybees (Scheiner et al., 1999, 2001a,b, 2003a, 2004, 2005; Behrends and Scheiner, 2012). We here reanalyzed data on olfactory and tactile conditioning in which individual responsiveness to sucrose of bees was determined prior to the conditioning phase (dataset 16, 17, see Section Materials and Methods). For both datasets we sorted animals into subgroups of equal size defined by 4 ranges of individual GRSs. Subgroups with higher GRS reached higher asymptotes of average CR probability than subgroups with lower GRS (Chi-square, *p* < 0.001, Figures 6A,C, compare with Figures 1, 2 in Scheiner et al. (2001a) in which learning performance was measured by acquisition scores).

At the level of individuals we found that animals with higher GRS respond with higher stability to the CS than animals with lower GRS (Chi-square, *p* < 0.01, dataset 16, 17, Table 2).We also found a smaller percentage of non-responding animals at higher GRS (dataset 16, 17, Table 2, Figures 6B,D), hence significantly more non-responders were found in data sets with lower GRS as compared to datasets with higher GRS (Chi-square, *p* < 0.001). Both for olfactory and tactile conditioning we found that all animals started to respond within the first few conditioning trials (Figures 6B,D), consistent with our previous results.

### A heterogeneous Rescorla–Wagner model captures the learning dynamics of honeybees

**Figure 7:**
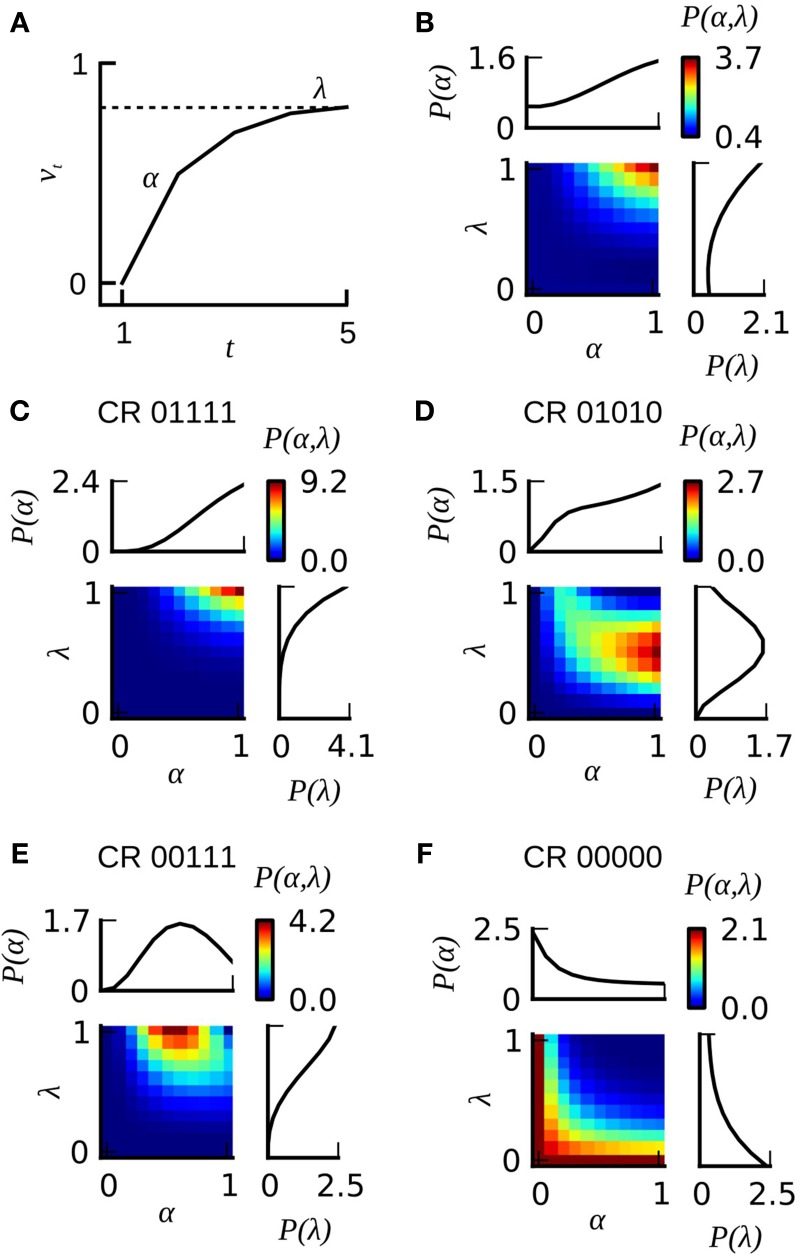
**The extended version of the Rescorla–Wagner model *RW*^*P*(α, λ)^. (A)** The gradual increase of associative strength *v*_*t*_ across training trials in an individual animal is described by two parameters: the rate of learning α and the asymptotic performance level λ. The probability for emitting a CR on a given trial is assumed to equal the associative strength. **(B)** Color-coded joint distribution *P*(α, λ) of learning parameters of all animals from dataset 21. Most animals have high learning rates and high learning asymptotes. The inlets on the top and on the right side of the colored joint distribution depict the cumulative probability distributions *P*(α) and *P*(λ) for which the joint distribution *P*(α, λ) has been summed over lambda or alpha, respectively. **(C–F)** Color-coded likelihoods for observing a particular sequence of CRs given different combinations of α and λ. The leftmost number of the CR sequence equals the CR in the first trial, and the rightmost number in the CR sequence equals the CR in the last trial.

The Rescorla–Wagner model (Rescorla and Wagner, 1972) provides a simple and yet influential theoretical account of associative learning during classical conditioning. Applying this theory in a straightforward way to any of our datasets from absolute conditioning allows us to estimate two parameters: the learning rate α and the US effectiveness λ (Section Materials and Methods, Equation 4). However, given the substantial degree of heterogeneity in learning performance, these two group-average parameters provided an invalid description of associative learning within the animals under investigation. In order to make the Rescorla–Wagner theory applicable to behavioral data in the honeybee, some learning heterogeneity has to be introduced in the formalism. For each animal in a given dataset we estimated the learning parameters separately (see Section Materials and Methods and Figure 6). For example, an animal with the CR sequence of 01111 will most probably have a high learning rate and a high asymptote, while an animal with a CR sequence of 00000 requires at least one of its two learning parameters to be near to zero (see Figures 7C,F). In order to estimate the total distribution of learning parameters *P*(α, λ) for a given dataset we summed and normalized the individual probability distributions. The total probability distribution *P*(α, λ) illustrates the learning heterogeneity in any given dataset (Figure 7B), and it also allow comparing the modulation of this distribution between different training conditions or experimental groups. As it can be shown by cross-validation, the assumption of a heterogeneous learning process can well describe the behavioral data, while on the other hand the assumption of a homogeneous learning process within the group must be rejected (Table 3, upper part). This was also true for datasets that consisted of individuals from a narrow range of GRSs (datasets 16, 17, Table 3, upper part).

**Table 3:** **Performance comparison between the homogeneous and the heterogeneous Rescorla–Wagner model**.

**Dataset**	***RW***	***RW*^*P*(α, λ^)**
10	82.5 (0)	73.8 (1)
11	32.4 (0)	19.1 (1)
12	40.5 (0)	32.1 (1)
13	86.4 (0)	73.1 (1)
14	69.4 (0)	53.8 (1)
15, 1 s	31.5 (0)	25.4 (1)
15, 2 s	29.1 (0)	21.6 (1)
15, 3 s	29.8 (0)	22.3 (1)
15, 6 s	25.2 (0)	17.3 (1)
15, 10 s	16.5 (0)	11.9 (1)
15, 16 s	18.3 (0)	15.3 (1)
16, GRS 10	34.6 (0)	23.9 (1)
16, GRS 8–9	43.4 (0)	26.6 (1)
16, GRS 5–7	35.7 (0)	21.1 (1)
17, GRS 10	52.9 (0)	39.6(1)
17, GRS 8–9	48.8 (0)	29.6 (1)
17, GRS 5–7	26.7 (0)	14.1 (1)
18	109.3 (0)	73.0 (1)
19	119.1 (0)	72.0 (1)
21	76.6 (0)	60.7 (1)
Score	0	20
**NON-RESPONDERS EXCLUDED**		
10	61.9 (1)	61.9 (0)
11	12.0 (0)	11.2 (1)
12	28.6 (0)	27.0 (1)
13	62.8 (0)	60.8 (1)
14	51.3 (0)	48.4 (1)
18	77.3 (0)	63.7 (1)
19	86.3 (0)	64.6 (1)
21	46.3 (1)	46.8 (0)
Score	2	6
**PERMUTED DATA**		
10	82.3 (1)	85.8 (0)
11	31.6 (1)	33.0 (0)
12	40.1 (1)	41.7 (0)
13	85.9 (1)	89.1 (0)
14	68.8 (1)	71.3 (0)
18	107.8 (1)	112.2 (0)
19	117.3 (1)	120.3 (0)
21	76.2 (1)	80.1 (0)
Score	8	0

The performance of the classical Rescorla–Wagner model RW as compared with an extended version RW^P(α,λ)^ that assumes a heterogeneity in US effectiveness lambda and learning rate alpha among individual animals within a dataset. For each model and data set, the mean negative log-likelihood on the test data was calculated after 500 rounds of four-fold cross-validation. Numbers in parenthesis indicate the performance rank of each model for a given dataset. The middle part of the table shows the comparison between the two models on data in which non-responding animals have been removed. The lower part of the table shows the comparison between the two models on surrogate datasets in which the CRs of the original data have been permuted across animals on each trial.

Since non-responding animals contributed a large part to the learning heterogeneity in the datasets, we asked if the assumption of a homogeneous learning process would be valid after non-responding animals were removed from the data. By cross-validation we found that in 6 out of 8 cases (Table 3, middle part) the data was still best described by a heterogeneous learning process. Hence even if non-responders are excluded from the data, the remaining animals are not adequately described by a single set of learning parameters rate α and λ. This finding is consistent with our data analysis, in which we found subtle differences in the response characteristics of responding animals (e.g., early responding animals respond with higher stability than late responding animals).

It should be noted, that when permuting the CRs in a dataset across individuals on each trial (as exemplified in Figure 1D for dataset 21), cross-validation reveals that the homogeneous Rescorla–Wagner model is always the most eligible model (Table 3, lower part). These surrogate binary datasets represent the case in which the group-average learning curve equals the response probability in individuals. However, this property was never observed in real data.

The heterogeneous version of the Rescorla–Wagner model is also consistent with our findings on memory retention in Experiment 1 and 2. Given the typical shape of the probability distribution *P*(α, λ) (see Figure 7B) it follows that animals with a CR history of 0(1), 01, or 0111 will have a high retention score because these observations are most likely emitted by individuals with high values of α and λ. On the other hand, animals with a CR history of 001 or 0001 will show lower retention scores because these observations are most likely emitted by animals with lower learning parameters α and λ.

## Discussion

Group-average learning curves confound three behavioral parameters of individual learning in the honeybee: (1) the ability to learn a task (as indirectly observed in the percentage of non-responders), (2) the latency in trial time until the first response is initiated (*t*_*firstCR*_), and (3) the stability of subsequent responses (CR stability). Here we show that these three parameters are useful measures to disentangle the group-average learning performance from individual learning. The group-average perspective implied that honeybees required at least three conditioning trials to reach asymptotic levels of CRs. However, these dynamics were not supported at the individual level. The majority of animals that showed any response at all already extended their probosces in the second trial, i.e., after the experience of a single CS-US pairing. Once having responded for the first time, honeybees continued to respond with a high probability during training as well as in the retention test, irrespective of the number of experienced conditioning trials. In summary, three conclusions can be drawn: (1) The gradual rise in the group-average learning performance during the first few trials primarily reflects an increasing proportion of responding animals. It does not however provide direct evidence for a gradual performance increase in individuals. (2) The asymptote of the group-average learning curve mostly reflects the proportion of responding animals in the population, but does not represent a performance maximum in individuals. (3) Memory retention in individuals resembles a constant continuation of the behavior expressed in the conditioning phase. Consequently, group-average memory retention mostly reflects the proportion of individuals that showed a CR during training. However, it does not provide a measure of memory strength across individuals within the population.

### A strict distinction between single-trial and three-trial induced 24 h memories is not supported by individual behavior and biochemical analysis

The effect of the number of conditioning trials on memory formation in the honeybee has been studied in behavior and biochemistry (for reviews see Menzel, 1999; Müller, 2012). The current model predicts lower and less odor-specific 24 h group-average memory retention after single-trial than after multiple-trial conditioning (Menzel, 1990, Figure 9.8; Müller, 2012, Figure 1; Menzel, 2012, Figure 2; Lefer et al., 2013). However, in Experiments 1 and 2 we could not find evidence that in individuals 24 h memory retention probability or odor-specificity was enhanced after more training trials (Figure 4). We suggest that the population-level finding of higher memory retention after multiple-trial conditioning primarily reflects an increased proportion of learners as compared to single-trial conditioning. After one conditioning trial typically at most half of the animals in a given conditioning group show a stable CR, which only leads to moderate retention scores when the whole group is tested. On the other hand, during three-trial conditioning typically most of the animals that are able to learn the task start to show a stable CR, which results in high group-average memory retention. The single-animal perspective could also provide an explanation for a controversial finding by Sandoz et al. (1995), showing that honeybees can form a life-long memory even after a single conditioning trial. Group-average memory retention after single-trial conditioning depends on the distribution of the learning speed across the population. It seems reasonable to assume that this distribution was skewed toward high learning rates under the experimental conditions in this study (see Figure 1 in Sandoz et al., 1995), and that consequently group-average retention did not differ after single-trial and three-trial conditioning.

Equal memory performance after single- and three-trial induced memories, however, does not necessarily imply equal underlying neuronal mechanisms. Indeed, there are several studies showing, that single-trial and multiple-trial induced memories differ fundamentally in the molecular mechanisms of their consolidation (Müller, 2002; Eisenhardt, 2006; Schwärzel and Müller, 2006): One conditioning trial is thought to produce a short-term memory (STM) which is independent of gene transcription and translation, while multiple-trial conditioning induces long-term memories that can be pharmacologically dissected into a translation sensitive 24 h memory (e-LTM) (Friedrich et al., 2004; Stollhoff et al., 2005) and a transcription and translation sensitive memory which lasts 3 or more days (l-LTM) (Grünbaum and Müller, 1998; Wüstenberg et al., 1998; Lefer et al., 2013).

We could not reproduce the finding that three-trial induced 24 h-memory is translation-dependent (Friedrich et al., 2004; Stollhoff et al., 2005) (Figure 5). There are several other studies which also found that multiple-trial induced 24 h memory retention is neither affected by interfering with translation (Menzel et al., 1993; Wittstock et al., 1993; Wüstenberg et al., 1998) nor by interfering with the PKA signaling pathway that leads to translation (Matsumoto et al., 2014).

Thus, whether one finds a translation-dependency of 24 h memory retention or not might depend on the experimental conditions. Which experimental factors could affect the translation-dependency of 24 h memory? One factor could be the satiation level of the animals, as three-trial induced 24 h memory is translation-dependent in bees which were fed 18 h before conditioning but not in bees which were fed 4 h before conditioning (Friedrich et al., 2004). Another factor could be contextual changes during the conditioning procedure, i.e., when animals are removed from the conditioning setup between training trials, as such contextual changes can affect learning performance (Gerber et al., 1998). Moreover, differences in 24 h memory performance and its sensitivity to pharmacological treatments could be due to different time windows during which the translation inhibitors take effect. Multiple-trial induced 3- and 4-day memories, for example, require gene transcription during two separate time windows, one during conditioning (Lefer et al., 2013) and another one 3–8 h after conditioning (Wüstenberg et al., 1998; Lefer et al., 2013). Accordingly, studies which failed to show a translation-dependency of single-trial and multiple-trial induced 24 h memories might have missed the critical time window during which translation is required. On the other hand, there are several studies, which indicate that 24 h memories already depend on gene transcription: pharmacological interference with histone acetylation or DNA methylation, two epigenetic processes that regulate transcription, affect 24 h memories after single-trial conditioning (Merschbaecher et al., 2012) and multiple-trial conditioning (Biergans et al., 2012; Merschbaecher et al., 2012; Lockett et al., 2014).

To conclude, it appears that the molecular mechanisms of single-trial induced memories as well as of 24 h memories are not fully understood yet, and the strict separation of single-trial induced, translation-independent STM, multiple-trial induced, translation-dependent e-LTM, and transcription-dependent l-LTM might not be as strict as is commonly assumed. In order to shed light on the molecular basis of single-trial induced memories it might be helpful to investigate how individual behavioral performance relates to molecular processes of learning and memory consolidation by introducing individual behavior in the analysis. For example, one may compare differences in PKA levels or other molecular signatures of memory consolidation in animals with a CR history of 0(1) and 0111 in order to elucidate the effect of training intensity on the neuronal substrate in responding animals. It would also be informative to check for differences between responding and non-responding animals in order to elucidate whether non-responders are in fact non-learners. In this context it should be noted that several recent studies in the honeybee enhanced their analysis of neuronal activity by taking into account individual behavior (Roussel et al., 2010; Rath et al., 2011).

### Implications for future research

Several factors, such as satiation level (Page et al., 1998; Ben-Shahar and Robinson, 2001; Scheiner et al., 2003b; Friedrich et al., 2004), behavioral role (Scheiner et al., 1999, 2001b, 2003a), season (Scheiner et al., 2003a), or age (Behrends and Scheiner, 2012) affect sucrose responsiveness in the honeybee, which in turn can affect learning performance. A sample of wild-type honeybees caught at the entrance to the hive will naturally consist of individuals that vary in several if not all of these factors. While satiation levels can be calibrated by standardized feeding routines before the start of an experiment (Friedrich et al., 2004), the sample will still contain an unknown composition of different types of foragers at different ages, which may cause inexplicable variability in learning performance when different experimental groups are compared (Behrends et al., 2007; Scheiner and Amdam, 2009; Münch et al., 2010). Experimentally one can control for the actual learning abilities of individuals in a given sample by testing honeybees for their responsiveness to sucrose prior to conditioning, or by selecting animals based on their performance in a preconditioning phase (Chandra et al., 2010). Complementary to this experimental approach, we suggest that analyzing data at the single-animal level may help to resolve the problem of inter- and intra-group variability, as often encountered in classical conditioning of the PER (Frost et al., 2012; Matsumoto et al., 2012). Our analysis showed that an early and stable CR was the most salient and invariable behavioral feature of individual learning during standard training conditions. This finding may be exploited by explicitly studying the effect of altered training parameters or *in vivo* pharmacological (Schwärzel and Müller, 2006; Felsenberg et al., 2011) or epigenetic interventions (Lockett et al., 2010; Biergans et al., 2012) on this behavioral performance benchmark.

Computational models of plasticity in the insect brain have not been constrained by individual learning dynamics to date. In Drosophila, a long-held notion exists that the expression of behavior in individuals follows the group-average (Quinn et al., 1974), and only recently has this issue been touched on again (Chabaud et al., 2010). Consequently theoretical studies tend to rely on group-average performance, as for example observed in a final test phase after aversive classical conditioning in the T-maze (Young et al., 2011; Wessnitzer et al., 2012). Our study described several characteristics of associative learning in individual honeybees, and yet more data from different classical conditioning protocols may be shared by other laboratories. Integrating these behavioral constraints into current models of plasticity in the insect brain is the focus of ongoing research.

### Conflict of interest statement

The authors declare that the research was conducted in the absence of any commercial or financial relationships that could be construed as a potential conflict of interest.
